# Supplementary material for: Oxidative stress and the presence of bacteria increase gene expression of the antimicrobial peptide aclasin, a fungal CSαβ defensin in Aspergillus clavatus
Source: PeerJ. 2019 Feb 25;7:e6290. doi: 10.7717/peerj.6290 (PMC6394349; doi:10.7717/peerj.6290)
Supplement: Supplemental Information 1 [file peerj-07-6290-s001.docx]

| **Target gene** | **Primer sequence (5’ - 3’)** | **GenBank accession number** | **Efficiency (%)** |
| --- | --- | --- | --- |
| *act1*  (ACLA_095800) | F TTCCATTGTCGGTCGTCCC | XM_001270072.1 | 102.5 |
|  | R GTGCCTCATCACCGACATAG |  |  |
| *aclasin* (ACLA_006820) | F CAGGCTTGCCAATATACGAGC | XM_001273349.1 | 102 |
|  | R GGACATGGGTGTGGAGGATG |  |  |
| *cat2*  (ACLA_044200) | F GACTGGGAGCTGACCAAGAG | XM_001275125.1 | 100.5 |
|  | R CGCGAGATTTTCTCGTAAGC |  |  |
| *hsp30*  (ACLA_088240) | F CACCAAAGTTCGATGTGTGC | XM_001272560.1 | 95.5 |
|  | R CTCGGAGTTGGTTTTGCTGT |  |  |

F: Forward, R: Reverse.
