# Supplementary material for: Oxidative stress and the presence of bacteria increase gene expression of the antimicrobial peptide aclasin, a fungal CSαβ defensin in Aspergillus clavatus
Source: PeerJ. 2019 Feb 25;7:e6290. doi: 10.7717/peerj.6290 (PMC6394349; doi:10.7717/peerj.6290)
Supplement: Supplemental Information 2 [file peerj-07-6290-s002.docx]

| **Putative fungal CSαβ defensin** | **Origin** | **Gene** | **GenBank accession number (analysed region) ^a^** |
| --- | --- | --- | --- |
| Aclasin  (Zhu, 2008) | *Aspergillus clavatus* | *aclasin*  (ACLA_006820) | NW_001517102.1  (438950 - 440450) |
| Afusin  (Zhu, 2008) | *Aspergillus fumigatus* | *afusin*  (AFUA_7G05180) | NC_007200.1  (1221284 -1222784) |
| Aflasin-1  (Zhu, 2008) | *Aspergillus flavus* | *aflasin-1*  (AFLA_065050) | NW_002477244.1  (224557 - 226057) |
| Agysin  (Zhu et al., 2012) | *Nannizzia gypsea* | *agysin*  (MGYG_04330) | NW_003345199.1  (2391417 - 2392917) |
| Aflasin-3  (Zhu, 2008) | *Aspergillus flavus* | *aflasin-3*  (AFLA_072140) | NW_002477237.1  (21413 - 22913) |
| Acasin  (Zhu, 2008) | *Histoplasma capsulatum* | *acasin*  (HCAG_07321) | NW_001813975.1  (1207440 - 1208940 |
| Nefisin-2  (Zhu, 2008) | *Neosartorya fischeri* | *nefisin-2*  (NFIA_026320) | NW_001509763.1  (544984 - 546484) |
| Vedasin  (Zhu et al., 2012) | *Verticillium dahliae* | *vedasin*  (VDAG_04103) | ABJE01000630  (82327 - 83827) |

^a^ 1,500 bp upstream the gene.
