# Supplementary figures and images for: Oxidative stress and the presence of bacteria increase gene expression of the antimicrobial peptide aclasin, a fungal CSαβ defensin in Aspergillus clavatus
Source: PeerJ. 2019 Feb 25;7:e6290. doi: 10.7717/peerj.6290 (PMC6394349; doi:10.7717/peerj.6290)

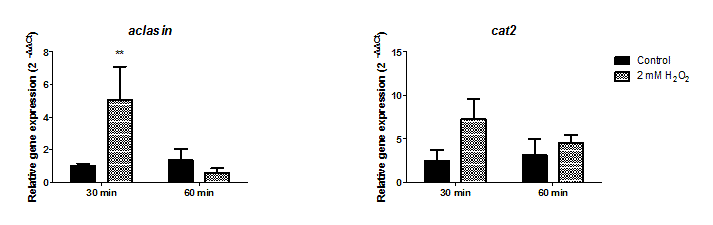

Supplement: Supplemental Information 3 [file peerj-07-6290-s003.png]

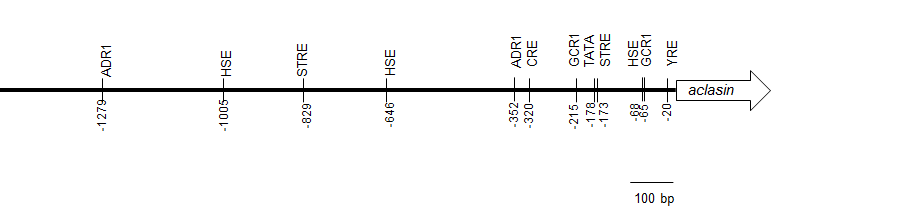

Supplement: Supplemental Information 4 [file peerj-07-6290-s004.png]

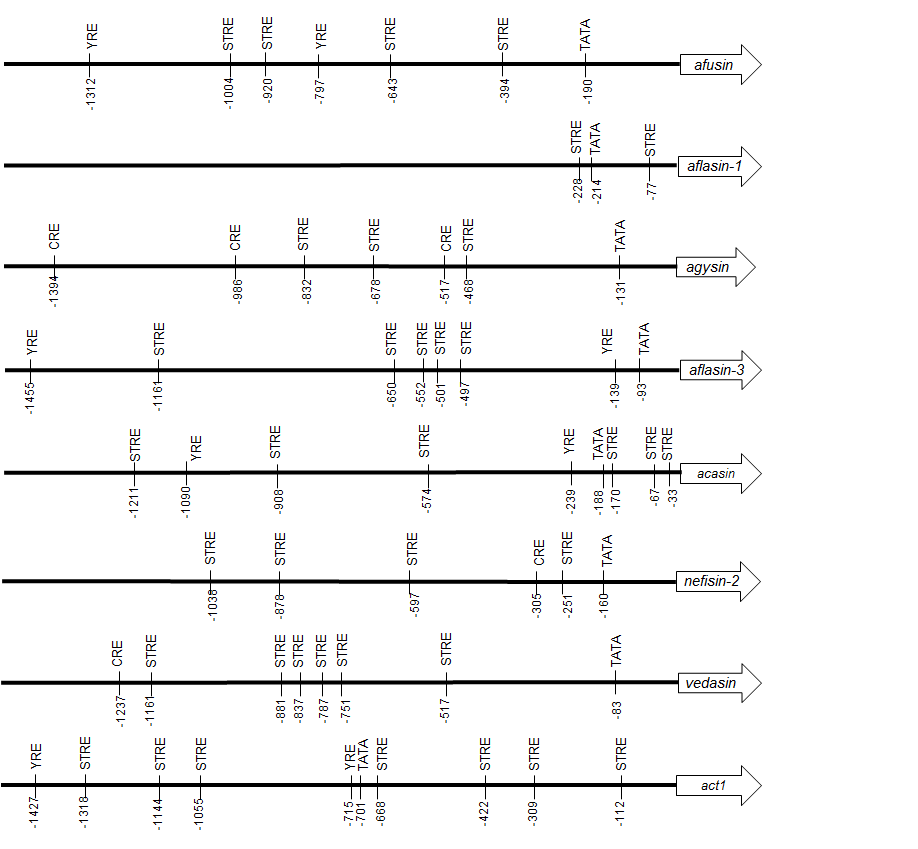

Supplement: Supplemental Information 5 [file peerj-07-6290-s005.png]
